# Betatron radiation and emittance growth in plasma wakefield accelerators

**DOI:** 10.1098/rsta.2018.0173

**Published:** 2019-06-24

**Authors:** P. San Miguel Claveria, E. Adli, L. D. Amorim, W. An, C. E. Clayton, S. Corde, S. Gessner, M. J. Hogan, C. Joshi, O. Kononenko, M. Litos, W. Lu, K. A. Marsh, W. B. Mori, B. O'Shea, G. Raj, D. Storey, N. Vafaei-Najafabadi, G. White, Xinlu Xu, V. Yakimenko

**Affiliations:** 1 LOA, ENSTA ParisTech, CNRS, Ecole Polytechnique, Institut Polytechnique de Paris, 91762 Palaiseau, France; 2 University of Oslo, NO-0316 Oslo, Norway; 3 Stonybrook University, Stony Brook, NY 11794, USA; 4 University of California Los Angeles, Los Angeles, CA 90095, USA; 5 CERN, Geneva, Switzerland; 6 SLAC National Accelerator Laboratory, Menlo Park, CA 94025, USA; 7 University of Colorado Boulder, Boulder, CO 80309, USA; 8 Tsinghua University, Beijing 10084, People's Republic of China

**Keywords:** plasma, accelerators, particle beams, radiation, gamma-rays

## Abstract

Beam-driven plasma wakefield acceleration (PWFA) has demonstrated significant progress during the past two decades of research. The new Facility for Advanced Accelerator Experimental Tests (FACET) II, currently under construction, will provide 10 GeV electron beams with unprecedented parameters for the next generation of PWFA experiments. In the context of the FACET II facility, we present simulation results on expected betatron radiation and its potential application to diagnose emittance preservation and hosing instability in the upcoming PWFA experiments.

This article is part of the Theo Murphy meeting issue ‘Directions in particle beam-driven plasma wakefield acceleration’.

## 1. Introduction

Plasma wakefield acceleration (PWFA) is a method for accelerating charged particles using large electric fields sustained by plasma waves (up to hundreds of GV m^−1^ for the accelerating longitudinal field) [1]. In this scheme, a relativistic electron bunch (called *drive* bunch) is sent through the plasma, exciting perturbations in the plasma density that forms a plasma wave. A second electron bunch (or *trailing* bunch) can then be injected at the accelerating phase of this plasma wave, receiving a substantial gain in energy.

When the drive bunch density is significantly greater than the plasma density, all free plasma electrons are expelled out from the beam propagation axis, creating a positively charged ion cavity behind. This regime is called the *bubble* or *blow-out* regime [2–4], and it is considered to be one of the most suitable regimes for electron acceleration. This nonlinear regime allows acceleration of electrons using large accelerating gradients (typically an order of magnitude larger than in the linear regime, where the density perturbation behind the drive bunch does not reach 100%), and has an ideal field structure for preserving the quality of an electron beam during its acceleration [5] and for reaching high energy transfer efficiencies from the drive to the trailing beam [6]. But to reach this blow-out regime, the drive beam needs to have extreme parameters: very high bunch density and beam size and bunch length of the order or smaller than the plasma wavelength. FACET is one of the only facilities in the world to provide an electron beam of this kind, where in 2016 a 9 GeV energy gain in a beam-driven PWFA was experimentally demonstrated [7].

Such large accelerating fields make this technique a promising alternative to conventional accelerators based on radio-frequency (RF) cavities, whose maximum acceleration gradient can be several orders of magnitude below the typical accelerating gradients of PWFA. However, in terms of beam quality, the PWFA scheme has not yet reached the same performance as conventional acceleration technique. Beam quality preservation is one of the most important issues to overcome for most of the PWFA applications, such as the PWFA-based linear collider. This new milestone of PWFA will be explored at the future FACET II facility [8], which is expected to produce unprecedented electron beams, in particular in terms of beam current, spot size and beta function at focus, bunch length and emittance.

Several processes might deteriorate the quality of an electron beam during acceleration. In this article, we will focus on the emittance growth caused by a mismatched propagation of the beam in the plasma and by the presence of the hosing instability. These processes are expected to be the most relevant for beam quality degradation in the actual state of PWFA experiments. Furthermore, they are both difficult to measure experimentally *in situ* with a non-destructive method. As shown in this article, a possible non-destructive diagnostic for these processes would be the use of the betatron radiation emitted by the beam electrons in the ion cavity. The use of betatron radiation as a diagnostic of plasma accelerators was studied in laser wakefield accelerators and was successfully applied to infer electron beam size in the bubble [9–11] and to study injection mechanisms [12].

## 2. Betatron radiation at FACET II: simulation results

The betatron radiation in PWFA accelerators is emitted by the drive and trailing electron bunches due to the transverse forces present in the ion cavity acting upon the electrons. These forces are proportional to the transverse displacement of the electrons with respect to the propagation axis and result in oscillating trajectories, called betatron oscillations. The spatial period of the electron oscillation is called the betatron wavelength given by $\lambda_{\beta }=\sqrt{2\gamma }\lambda_{p}$ in the blow-out regime, where λ_*p*_ is the plasma wavelength and *γ* the Lorentz factor of the electron. For conditions relevant to FACET II, the radiation emitted by an electron following this type of trajectory is very similar to the radiation produced in high-*K* wigglers, i.e. very collimated in the forward direction (*θ*≃*K*/*γ*≪1) and with a synchrotron-like photon energy spectrum [13]. This is the so-called betatron radiation [13–15].

When the beam is said to be ‘matched’, its spot size remains constant during its propagation in the plasma. The conditions for matching are best expressed using the Twiss parameters, defined for the *x* direction as

$$\alpha_{x}=-\frac{\left\langle xx^{\prime }\right\rangle }{\epsilon_{x}},\;\beta_{x}=\frac{\left\langle x^{2}\right\rangle }{\epsilon_{x}}\;\mathrm{and}\;\gamma_{x}=\frac{\left\langle x^{\prime 2}\right\rangle }{\epsilon_{x}}.$$

The Twiss parameters of the bunch need to be tuned in order to obtain a matched propagation, and in the blow-out regime, the matched Twiss parameters read *β*_ matched_ = λ_*β*_/2*π* and *α*_matched_ = 0 [16,17]. When matching conditions are not met, individual oscillations of the electrons give rise to beam envelope oscillations: the spot size (or RMS radius) of the beam will oscillate while propagating in the plasma [17,18].

**Figure 1.**
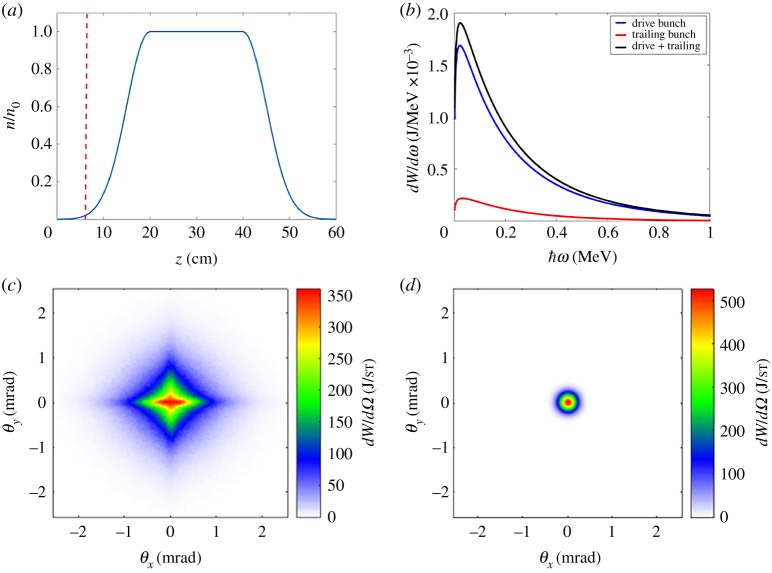
(*a*) Plasma density profile used for QuickPIC simulations (*n*_0_ = 4 × 10^16^ cm^−3^). Red-dashed line shows the position of the focal plane of the trailing bunch for the matched conditions. (*b*) Photon energy spectrum of the radiation emitted by the trailing bunch (red), drive bunch (blue) and both (black). (*c*) Radiation angular distribution of the drive bunch (J/sr). (*d*) Radiation angular distribution of the trailing bunch (J/sr).

FACET II will deliver electron bunches with optimal parameters, such as high current, small spot size and beta function at focus, small bunch length and low emittance, for the next generation of PWFA experiments. In the two bunch configuration, the drive bunch will excite a plasma wave in the pre-ionized lithium vapour target, and the trailing bunch will propagate behind the drive bunch, experiencing the accelerating and focusing fields of the plasma wave. The plasma density profile used in the simulations corresponds to the expected vapour density profile of the lithium oven [19,20] that will be used as the plasma target in the experiment. The coordinate system used in our simulations is such that *z* is the longitudinal coordinate corresponding to the propagation direction of the electron bunch, *x* and *y* are the transverse coordinates forming a right-handed Cartesian coordinate system. Taking *z* = 0 as the beginning of the simulation, the simulated plasma density profile consists of a semi-Gaussian up-ramp with maximum at *z* = 20 cm, a plateau region (*n*_0_ = 4 × 10^16^ cm^−3^) from *z* = 20 cm to *z* = 40 cm, followed by a semi-Gaussian down-ramp from *z* = 40 cm to *z* = 60 cm. Parameters of the drive and trailing electron bunches used in the simulations are showed in table 1 and the plasma density profile is plotted in figure 1*a*.

**Table 1.** Relevant beam parameters at the location of the *x* and *y* trailing waist used in the simulations: *α* and *β* are the Twiss parameters, *Q* is the beam charge, *σ*_*z*_ is the RMS bunch length, *E* is the energy, *ϵ*_*N*_*x*,*y*__ is the normalized emittance, Δ*z* is the longitudinal separation distance between the bunches and Δ*W*_*x*,*y*_ is the distance between locations of the drive waist and the trailing waist (Δ*W*_*x*_ for *x* waist, Δ*W*_*y*_ for *y* waist).

	drive beam	trailing beam
*α*_*x*,*y*_	4.2, 1.6	0, 0
*β*_*x*,*y*_ [m]	0.7, 0.7	0.05, 0.05
*Q* [nC]	1.6	0.5
*σ*_*z*_ [μm]	6.4	2.8
*E* [GeV]	10	10
*ϵ*_*N*_*x*,*y*__ [mm · mrad]	3.4, 3.0	3.2, 3.2
Δ*z* [μm]	150	
Δ*W*_*x*,*y*_ [cm]	16, 31	

In order to explore potential use of the betatron radiation in PWFA accelerators to assess beam quality deterioration, we simulated using the QuickPIC code [21,22] the expected FACET II electron bunches passing through the aforementioned plasma target, and then output the trajectories of the electrons to numerically compute emitted radiation using the Lienard–Wiechert fields [23]. Since the electrons are highly relativistic and the strength parameter *K* of the betatron oscillations [13] is large compared to 1, we used the synchrotron approximation to describe the angular and spectral distribution of the betatron radiation. Figure 1*b* shows the photon energy spectrum and figure 1*c*,*d* shows the angular distributions of the betatron radiation emitted by both bunches when the focal plane of the trailing bunch is set at *z* = 6.3 cm, shown as a red dashed line in figure 1*a*. These plots show typical values of divergence (∼ mrad), total radiated energy (∼ mJ) and gamma-ray spectrum for the FACET II beam parameters.

**Figure 2.**
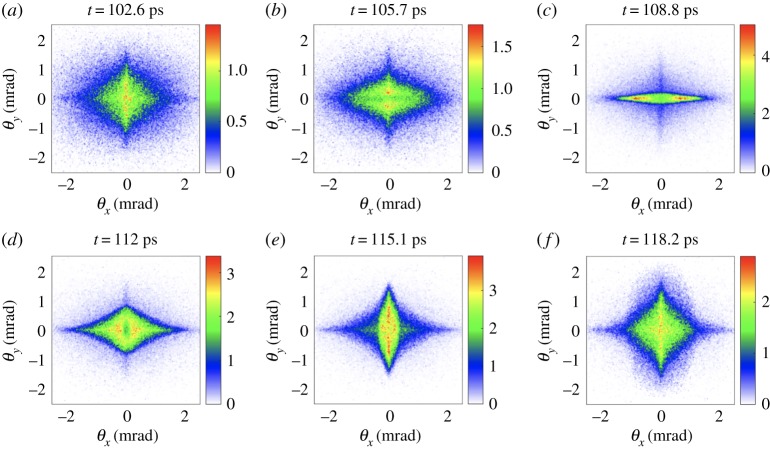
Betatron radiation angular distributions in J/sr emitted by the drive bunch at six consecutive timesteps. The timesteps represented here are *t* = 102.6, 105.7, 108.8, 112, 115.1 and 118.2 ps, corresponding to the beginning of the plateau region (*z* = 20 cm). In this simulation, the trailing focal plane position is at *z*_trailing_ = 12 cm, the *x* drive focal plane position is at *z*_drive, *x*_ = *z*_trailing_ + Δ*W*_*x*_ = 28 cm and the *y* drive focal plane position is at *z*_drive, *y*_ = *z*_trailing_ + Δ*W*_*y*_ = 43 cm. (Online version in colour.)

The difference between the angular distribution of the radiation emitted by the drive bunch and the trailing bunch is related to beam parameters of each bunch. As mentioned above, if the matching conditions are not met, beam envelope oscillations are present during the propagation of the bunch in the ion cavity. These envelope oscillations are described by the following differential equation:

$$\sigma_{i}^{\prime \prime }+k_{\beta }^{2}\sigma_{i}-\frac{\epsilon_{i}^{2}}{\sigma_{i}^{3}}=0,$$

where the derivatives are with respect to the longitudinal coordinate *z*, *σ* is the spot size, $k_{\beta }=2\pi /\lambda_{\beta }=k_{p}/\sqrt{2\gamma }$ with *k*_*p*_ the wavenumber associated with the plasma frequency, *ϵ* is the geometrical emittance and *i* represents the transverse coordinate *x* or *y*. When a beam is azimuthally symmetric both spot sizes *σ*_*x*_ and *σ*_*y*_ oscillate in phase, so that the electron beam preserves its azimuthal symmetry during propagation. This is the case for the FACET II trailing beam and leads to an azimuthal symmetry in the radiation angular distribution. If the beam parameters are not symmetric in the transverse directions, as for the FACET II drive beam, the transverse spot sizes evolve differently, leading in some cases to not-in-phase envelope oscillations, so that the transverse profile of the bunch oscillates between a horizontal and a vertical ellipse. These not-in-phase envelope oscillations, as shown in figure 2, affect the radiation angular distribution. This figure shows asymmetric betatron radiation angular distribution emitted in six consecutive timesteps separated by 3.1 ps, in which we can observe the transition from a horizontal ellipse (figure 2*c*) to a vertical ellipse (figure 2*e*). We note here that while we have access in simulations to the time evolution of the betatron radiation, in experiments the betatron measurements generally provide time-integrated angular profiles (figure 1*c*,*d*).

## 3. Betatron diagnostics: simulation results

Beam quality preservation in PWFA is one of the most important aspects to be experimentally proven in future PWFA research. Here, we present the simulation results that demonstrate ability to use betatron radiation to detect mismatched propagation and beam centroid oscillation which lead to emittance growth and hosing instability.

### (a) Mismatched propagation and emittance growth

**Figure 3.**
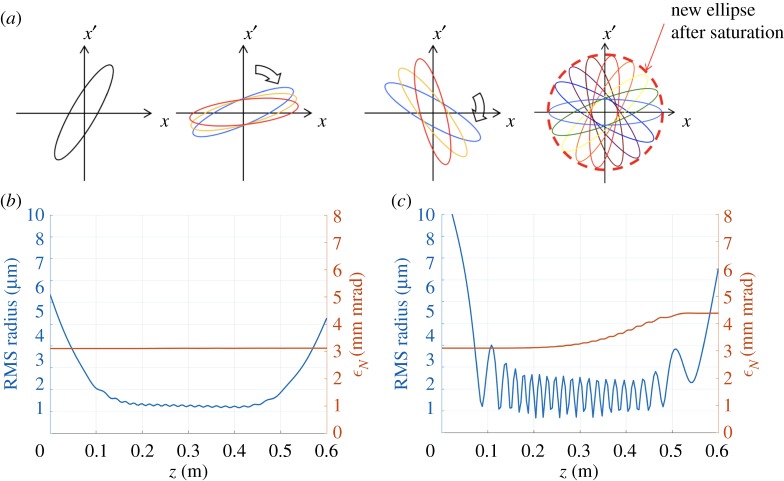
(*a*) Trace-space ellipse evolution for different energies (colours), red dashed circle illustrates geometrical emittance saturation. (*b*) and (*c*) RMS radius (in blue) and mean normalized emittance ($\sqrt{\epsilon_{Nx}\cdot \epsilon_{Ny}}$, in orange) for matched (*b*) and mismatched (*c*) cases.

For many applications of the PWFA technique, especially for the plasma-based linear collider, normalized transverse emittance must be kept constant during the acceleration process. Emittance growth in current PWFA accelerators is caused mainly by mismatched propagation in the plasma and chromatic effects due to the finite energy spread: trace-space ellipses of electrons with different energies rotate at a different rate in the trace space (*x*, *x*′) (or equivalently in the phase space (*x*, *p*_*x*_), with *x*′ = *p*_*x*_/*p*_*z*_), leading to an increase of the emittance (figure 3*a*) [16]. When the ellipses for each energy are circular^1^ instead of elliptical, even if individual particles would still describe circular orbits in trace space, the overall distribution remains the same as the beam propagates in the plasma. This beam matching leads to a constant spot size and allows mitigation of the emittance growth (figure 3*b*,*c* for comparison between matched and mismatched cases and associated emittance evolution).

The matched beta function *β*_matched_ = λ_*β*_/2*π* for a trailing beam inside an ion cavity can be determined for a given plasma density, since the betatron wavelength λ_*β*_ depends on plasma density. In experiments, plasma density is not uniform, and usually the electron beam goes through an up-ramp and down-ramp, which complicates prediction of matching conditions for a given plasma profile. It is then important for experiments to know when the beam is matched in the plasma, and betatron diagnostics can be a powerful tool to do this.

**Figure 4.**
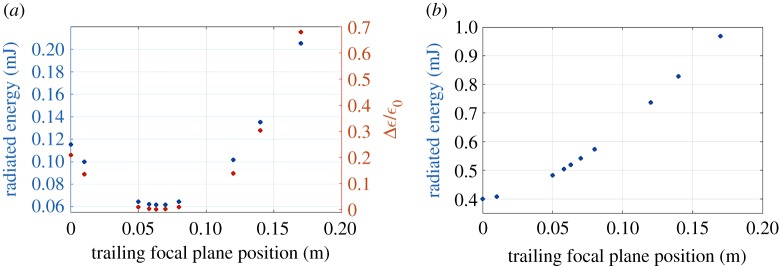
(*a*) Total radiated energy (blue) and relative emittance growth (orange) from the trailing bunch as a function of the focal plane position of the trailing bunch. (*b*) Total radiated energy from the trailing and drive bunches.

We run several simulations for the expected FACET II beam and plasma parameters for different focal plane positions of the trailing bunch inside the plasma. The drive bunch focal plane was also shifted consistently, so that different simulations correspond to different tunings of the final focusing magnets. In all these simulations we measured the emittance growth and correlated results with simulated betatron radiation (computed by post-processing the QuickPIC electron trajectories using the Lienard–Wiechert fields and the synchrotron approximation). The results are shown in figure 4*a*: we observe that the trailing beam is matched in the flat-top region when its focal plane is at *z* = 6.3 cm (red dashed line in figure 1*a*). When we either increase or decrease this distance, the trailing beam is not matched and this is translated into beam envelope oscillations and—as a consequence—an increase of the emittance (figure 3*c*). In these simulations, the trailing bunch has zero energy spread initially, but acquires a finite energy spread as it is accelerated in the plasma, which in turns lead to emittance growth depending on its matching to the plasma. The total betatron radiation emitted by the trailing bunch also has a minimum at 6.3 cm and it increases when we move apart from this focal position. This can be understood as follows: if the beam is not matched, individual electrons oscillate with a higher amplitude than in the matched case, so electrons radiate more energy. Thus this correlation shows that the betatron radiation emitted by an electron bunch in an ion cavity can be used to retrieve information about beam matching and the evolution of its emittance.

Experimentally, it is difficult to measure the radiation emitted by the trailing bunch separately from the radiation of the drive bunch. Figure 4*b* shows the energy radiated by the two bunches (drive + trailing) for the same values of trailing focal plane position as in figure 4*a*, and since the matching conditions for the drive bunch are not the same as for the trailing bunch we do not observe a minimum at *z* = 6.3 cm. This means that the measurement of the total radiated energy (drive + trailing) cannot be used directly to asses the matching of the trailing beam; the subtraction of the drive bunch radiated energy is required. This could be done by measuring first the betatron radiation of a single-bunch configuration (drive only), and then subtracting this ‘drive-only’ radiation from the total radiation emitted by the two bunches. Such measurements of the betatron radiation can be used to tune the experiment for trailing beam matching and emittance preservation.

### (b) Beam centroid oscillation and hosing instability

Hosing is a transverse instability that has been predicted theoretically to occur in the blow-out regime of PWFA [24,25] and studied experimentally [26]. This instability arises when longitudinally dependent transverse force acts upon the beam. Such a situation can occur when an electron beam is offset in a uniform ion column, which results in centroid oscillations with a growing amplitude. This instability yields to a large increase of the emittance and, if the instability grows enough, it might even cause the loss of portions of the bunch. Therefore, study and mitigation of this instability are very important for PWFA experiments.

**Figure 5.**
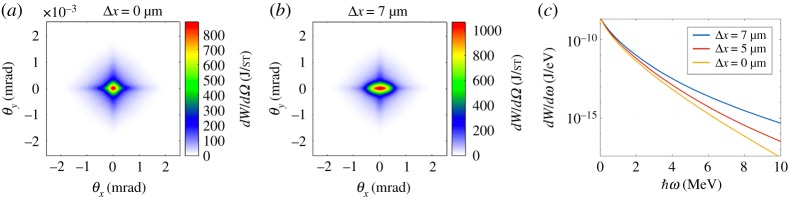
(*a*) Angular distribution (J/sr) of the betatron radiation emitted by both beams when no offset in the trailing was induced. (*b*) Angular distribution (J/sr) of the betatron radiation emitted by both beams when a 7 μm offset is introduced along the *x*-axis for the trailing bunch. (*c*) Energy spectrum of the betatron radiation emitted by both bunches for three different offset values. (Online version in colour.)

Our simulations show that betatron radiation could be exploited to also assess hosing instability. To simulate the hosing instability we introduced at the beginning of the simulations a small transverse offset in the trailing bunch. Figure 5 shows the effect of this offset on the angular distribution and energy spectrum of the betatron radiation. In figure 5*a*, we present the angular distribution of the betatron radiation emitted by both beams when no offset is introduced. Similar distribution is presented in figure 5*b* when an offset of 7 μm in the *x*-direction is introduced. We observe an increase of the radiated energy when an offset in the trailing beam is induced, and an increase in the divergence of the angular distribution in the direction where this offset was induced. Figure 5*c* shows the energy spectrum of the betatron radiation for three different values of the initial offset. A small difference at the tail of the distribution, at high photon energies, can be observed. In a similar way as for the emittance preservation, beam centroid oscillations leads to a larger oscillation amplitude of individual electrons, so that the total radiated energy also increases. These results demonstrate the possibility to use betatron radiation to fully characterize (in terms of direction and magnitude) an offset in the trailing beam which may seed hosing instability.

## 4. Conclusion and overlook

We presented simulation results of the expected betatron radiation properties for the future PWFA experiments at FACET II and the potential application of betatron radiation to investigate several processes occurring in PWFA. For FACET II electron beam and plasma target (lithium oven) parameters, the betatron radiated energy is expected to be of the order of the millijoule, with a milliradian divergence and a photon energy spectrum ranging from a few kiloelectronvolts up to the megaelectronvolt.

In our study, we demonstrated that the betatron radiation can be used to assess information about dynamics of the trailing electron beam propagating in a plasma wave, in particular regarding its matched or mismatched propagation and the beam centroid oscillations, which can lead to emittance growth and hosing. Thus, we conclude that betatron radiation is a powerful diagnostic to experimentally assess and mitigate emittance growth and hosing instability, which are of key importance for the next generation of PWFA experiments.

## Footnotes

1 Circular in the (*x*, *x*′) trace space where *x* is plotted in normalized units, so that *x*^2^ + *x*′^2^ = constant.

## Data accessibility

The data that support the findings of this study are available from the corresponding author upon request.
